# Comparison of nephrotoxicity of Colistin with Polymyxin B administered in currently recommended doses: a prospective study

**DOI:** 10.1186/s12941-018-0262-0

**Published:** 2018-03-23

**Authors:** Ritesh Aggarwal, Arun Dewan

**Affiliations:** 1 Department of Critical Care Medicine, Max Smart Super Speciality Hospital, Saket, New Delhi, India; 2 Department of Internal Medicine & Critical Care Medicine, Max Smart Super Speciality Hospital, Saket, New Delhi, India

**Keywords:** Colistin, Polymyxin B, Nephrotoxicity, Acute kidney injury

## Abstract

**Background:**

The most important concern with polymyxins (Colistin and Polymyxin B) use is nephrotoxicity. There is no prospective data comparing nephrotoxicity of these two drugs, when administered in high doses and as per current recommendations. We conducted a prospective study to compare their trend of nephrotoxicity in our patient population.

**Methods:**

Our study included adult ICU patients who received more than 48 h of Colistin or Polymyxin B and had no confounding factors for nephrotoxicity. Loading and maintenance doses were given as per a uniform protocol. Nephrotoxicity was defined as twofold increase in serum creatinine, or 50% decrease in estimated baseline creatinine clearance. Patients were followed up for 1 week after therapy. Statistical analysis was performed using SPSS version 20.0.

**Results:**

61 patients were included in Colistin group, and 51 patients in Polymyxin B group. Median Colistin dose was 233.3 (IQR 150–300) mg/day and median Polymyxin B dose was 200 (IQR 180–240) mg/day. Median duration of Colistin and Polymyxin B use was 7 (IQR 5–7) days and 7 (IQR 7–9) days respectively. Nephrotoxicity developed in 39.3% patients in Colistin group compared to 11.8% patients in Polymyxin B group. Mean onset of nephrotoxicity was 3.8 ± 0.8 days with Colistin, and 4.2 ± 0.7 days with Polymyxin B therapy. In bivariate analysis, Colistin daily dose ≥ 300 mg was found to be associated with nephrotoxicity. There was no effect of age or BMI on Colistin toxicity. Mean duration of renal failure was 4.9 ± 3.1 days with Colistin use, and 5.0 ± 2.4 days with Polymyxin B use. 75% patients in Colistin group and 83.3% patients in Polymyxin B group who developed nephrotoxicity recovered their renal function by 1 week.

**Conclusions:**

Colistin in currently recommended doses is significantly more nephrotoxic than Polymyxin B. Colistin toxicity is dose-dependent, mostly mild to moderate, and is reversible in most cases.

## Background

With the increasing incidence of multi-drug resistant (MDR) Gram negative infections, the focus has shifted to older antibiotics like polymyxins (Polymyxin E or Colistin and Polymyxin B) as agents of last resort [1]. Our recent understanding in the use of polymyxins is based on pharmacokinetic studies, which suggest a strategy of giving a loading dose, followed by higher intermittent doses at longer intervals [2, 3].

One of the major concern with the use of polymyxins is the risk of nephrotoxicity. The risk is further increased if they are used in higher doses. The reported rates of nephrotoxicity with polymyxins range from 15 to 60% [4–8]. Most of these studies have evaluated Colistin toxicity, and studies on Polymyxin B are very few. One of the reason for such a broad range of nephrotoxicity rates is the wide variation in doses of Colistin (and polymyxin) used in different studies. Also, most studies were retrospective and had a limited number of patients. There is no available data comparing nephrotoxicity of Colistin and Polymyxin B when they are used as per current recommendations. A recent prospective study [9] found Colistin to be significantly more nephrotoxic compared to Polymyxin B. However, most of the study patients in Colistin group and all the patients in Polymyxin B group did not receive a loading dose. The results were also confounded by use of second nephrotoxic drug in majority of patients.

The purpose of our study was to prospectively compare the nephrotoxicity of these two drugs, when used in optimum doses and explore associated risk factors in our patient population.

## Methods

### Design

We conducted a prospective, observational study from July 2016 to June 2017 in a 16-bed medical ICU, in a tertiary care hospital. The study was approved by Ethics committee and was granted a waiver for informed consent.

### Patients

We included all adult patients who received Colistin or Polymyxin B for more than 72 h. Exclusion criteria were (i) baseline CKD patient on hemodialysis or baseline creatinine clearance < 10 ml/min (estimated by Cockcroft Gault equation). (ii) Fluctuating renal parameters (increase or decrease in serum creatinine > 50% from baseline prior to starting polymyxins). (iii) Patient received intravenous contrast. (iv) Patient on any other nephrotoxic drug (aminoglycosides, vancomycin, amphotericin B, NSAIDs, cyclosporine, etc.). (v) Patient who received significant doses of diuretics (defined as furosemide > 20 mg or torsemide > 10 mg over 24 h). If the patient received two or more courses of polymyxins, only the first course was included in the study.

The choice of drug (Colistin vs Polymyxin B) was left to the treating physician. Once the drug was chosen, the loading and subsequent doses were given as per the institutional protocol (Table 1). Same brands of two drugs were used in all patients.

**Table 1:** Dosing schedule for Colistin and Polymyxin B

Dosing schedule for Colistin in adults		
Renal function	Loading dose	Maintenance dose
Normal renal function, or creatinine clearance > 50 ml/min	5 mg/kg (or 150,000 U/kg) IV over 40–60 min	2.5 mg/kg (or 75,000 U/kg) IV twice daily
Creatinine clearance 20–50 ml/min		2.5 mg/kg (or 75,000 U/kg) IV once daily
Creatinine clearance < 20 ml/min		2.5 mg/kg (or 75,000 U/kg) IV on alternate days
Patient on hemodialysis		Give additional dose of 50 mg (15,00,000 U) post dialysis
*Note*		
1. Use ideal body weight for Colistin dosing, and not actual body weight		
2. Maintenance doses are started 12 h after loading dose		

Dosing schedule for Polymyxin B in adults		
Loading dose: 2.5 mg/kg as 2 h IV infusion Maintenance dose: 1.5 mg/kg as 1 h IV infusion, twice daily		
*Note*		
1. Use actual body weight for Polymyxin B dosing		
2. Maintenance doses are started 12 h after loading dose		
3. There is no dose modification in renal failure		

### Measurements/data collection

In our study, nephrotoxicity or renal failure was defined as twofold increase in serum creatinine, or 50% decrease in estimated baseline creatinine clearance (Cockcroft Gault equation). Criteria were required to be fulfilled for at least 2 consecutive determinations 24 h apart, 48 h after starting Colistin or Polymyxin B. This corresponded to injury (I) class of RIFLE classification [10]. We didn’t include risk (R) class in our definition, as it is susceptible to various factors present in critically ill patients, and thus may not reflect the injury caused due to a nephrotoxic drug [11, 12]. We did not use urine output criteria to define nephrotoxicity. As suggested by acute dialysis quality initiative (ADQI) group, oliguria is a less consistent marker of acute kidney injury (AKI), compared to serum creatinine levels [10].

The association of different variables (age, gender, BMI, APACHE 2, DM, and dose) with nephrotoxicity was analysed. For the analysis of relationship between Colistin dose and nephrotoxicity, the daily dose being administered to patient at the time he met criteria for nephrotoxicity was used. Serum creatinine levels were measured daily for a minimum of 7 days after the course of drugs was over. Reversal of nephrotoxicity was defined as return of serum creatinine to less than or equal to 2 times of baseline creatinine or greater than 50% of baseline creatinine clearance.

### Statistical methods

The analysis of the data was done primarily by bivariate logistic regression to find the odds ratios of various factors, their confidence intervals and the statistical significance. Significance of the mean difference between groups for quantitative variables such as age, BMI and APACHE2 score was obtained by Student t-test after checking the equality of variances by Levene’s test. Student t-test was applied as our group sizes were sufficiently large. However, median and IQR were used for durations in view of their highly skewed distribution. In this case, the significance was tested by Wilcoxon test. Association between two qualitative factors was assessed using Chi square test for sufficiently large cell frequencies (n) and by Fisher exact test for small cell frequencies. For all the tests, the significance level was 5%. Statistical analysis was performed using SPSS version 20.0 (IBM, USA).

## Results

A total of 210 patients were identified for eligibility. Of the 116 patients identified in Colistin (COL) group, 61 met inclusion criteria. Of the 94 patients identified in the Polymyxin B group (PMB), 51 met inclusion criteria. The primary reason for ineligibility in both groups were use of second nephrotoxic drug (like intravenous contrast, aminoglycosides, amphotericin B, and diuretics infusion) and presence of end stage renal disease (ESRD).

The site of infection was lungs in 66 (58.9%), urinary tract in 19 (17.0%), blood stream in 10 (8.9%) and abdomen in 9 (8.0%) patients. The pathogens were isolated in 77 (68.7%) patients and include Klebsiella (27.7%), Pseudomonas (17.0%), Acinetobacter (19.6%) and *E. coli* (4.5%). There was no growth on cultures in 35 (31.2%) patients (Table 2).

**Table 2:** Patient characteristics in 2 groups

Variable	Colistin (n = 61)	Polymyxin B (n = 51)	P value
Gender, male	39 (63.9)	36 (70.6)	0.456
Age (years)	64.3 ± 13.3	63.4 ± 17.1	0.769
Weight (kg)	73.2 ± 11.8	69.3 ± 13.4	0.106
BMI (kg/m^2^)	26.8 ± 4.3	25.0 ± 4.7	0.041
APACHE 2 score	20.8 ± 4.2	22.0 ± 4.6	0.167
Baseline creatinine clearance (ml/min)	60.8 ± 33.5	55.4 ± 31.6	0.382
DM	30 (49.2)	22 (43.1)	0.523
HTN	12 (19.7)	11 (21.6)	0.071
Use of vasopressors	39 (63.9)	28 (54.9)	0.381
Number of days of antibiotic use [median(IQR)]	7 (5–7)	7 (7–9)	0.221
Median daily dose (mg)	233.3 (150–300)	200 (180–240)	0.116
*Site of infection*			
Lungs	28 (45.9)	38 (74.5)	0.002
Urine	19 (31.1)	0 (0)	< 0.001
Bloodstream	5 (8.2)	5 (9.8)	0.766
Abdomen	5 (8.2)	4 (7.8)	0.945
Others	4 (6.5)	4 (7.8)	0.792
*Pathogens*			
Klebsiella	19 (31.1)	12 (23.5)	0.370
Pseudomonas	8 (13.1)	11 (21.6)	0.235
Acinetobacter	12 (19.7)	10 (19.6)	0.993
*E. coli*	4 (6.5)	1 (2)	0.241
No growth	18 (29.5)	17 (33.3)	0.664

Data are presented as n (%), mean ± SD or median (IQR)

There were no significant differences in two groups with respect to baseline demographics or underlying risk factors for nephrotoxicity (Table 1). Mean estimated creatinine clearance for Colistin (COL) and Polymyxin B (PMB) group were 60.8 ± 33.5 and 55.4 ± 31.6 ml/min respectively. The median daily dose was 233.3 mg (IQR 150–300) in COL group and 200 mg (IQR 180–240) for PMB group.

All patients in Colistin group received a loading dose followed by maintenance doses starting at 12 h, as per institutional protocol (Table 1). Doses were modified according to ideal body weight and estimated creatinine clearance (Cockcroft Gault equation). Serum creatinine values were measured daily and in case of acute kidney injury, doses were modified as per creatinine clearance estimation. Colistin was withheld only if patient developed significant metabolic acidosis or hyperkalemia or needed hemodialysis.

All patients in Polymyxin B group (PMB), similarly, received a loading dose followed by maintenance doses based on actual body weight. There was no dose modification as per estimated creatinine clearance. Polymyxin B was not used in suspected or confirmed cases of urinary tract infection (UTI), due to its poor penetration in urinary tract. Median drug duration was 7 (IQR 5–7) days for Colistin and 7 (IQR 7–9) days for Polymyxin B.

The overall incidence of nephrotoxicity in all patients was 26.8% (30 of 112 patients). The incidence of nephrotoxicity was significantly higher in Colistin group (39.3%) compared to Polymyxin B group (11.8%) (P = 0.001). If we compare the incidence of renal failure as per RIFLE criteria, the incidence of risk, injury, failure—all classes combined was 52.4% in Colistin group and 19.6% in Polymyxin B group (P < 0.001). Mean onset of renal failure was 3.8 ± 0.8 days in COL group and 4.2 ± 0.7 days in PMB group. Mean duration of kidney injury was 4.9 ± 3.1 days in COL group and 5.0 ± 2.4 days in PMB group. 9 patients (14.7%) in COL group required hemodialysis and drug discontinuation. In comparison, only 1 patient (2%) in PMB group required hemodialysis and drug discontinuation.

We followed up all the patients for 1 week after the completion of course of antibiotics or drug discontinuation to understand the trends of nephrotoxicity. At the end of 1 week, there was reversal of nephrotoxicity in 75% of patients (18 of 24) in COL group. In PMB group, there was reversal in 83.3% (5 of 6) patients who had developed renal failure. In other words, at the end of 1 week follow up, 9.8% patients in COL group had renal failure, compared to 2% patients in PMB group. The primary and secondary outcomes have been shown in Table 3.

**Table 3:** Primary and secondary outcomes

Outcome	Number of patients (%)		P value
	Colistin (n = 61)	Polymyxin B (n = 51)	
Nephrotoxicity	24 (39.3)	6 (11.8)	0.001
*RIFLE*			
All classes (R+I+F)	32 (52.4)	10 (19.6)	< 0.001
R	8 (13.1)	4 (7.8)	0.105
I	13 (21.3)	4 (7.8)	0.009
F	11 (18.0)	2 (3.9)	0.004
*Secondary outcomes*			
Need for dialysis	9 (14.8)	1 (2.0)	0.018
Need for stopping medication	9 (14.8)	1 (2.0)	0.018
Onset of AKI (mean ± SD)	3.8 ± 0.8	4.2 ± 0.7	0.373
Duration of AKI (mean ± SD)	4.9 ± 3.1	5.0 ± 2.4	0.916
Reversal of nephrotoxicity, n (%)	18 (75.0)	5 (83.3)	0.666

Data are presented as n (%), or mean ± SD

The subgroup analysis of Colistin group has been shown in Table 4. Among the patients of this group, patients who received a daily dose of ≥ 300 mg (or ≥ 9 million international units) had higher incidence of renal failure (17 of 27, 63.0%) (P = 0.001) (OR 6.56). We also observed that incidence of renal failure was significantly less in patients whose baseline creatinine clearance was low, i.e. < 50 ml/min (5 of 28, 17.9%) than in patients with creatinine clearance ≥ 50 ml/min (19 of 33, 57.6%) (P = 0.002). The post hoc analysis of these patients revealed that patients with lower creatinine clearance received less daily dose of Colistin (150 mg, IQR 150–233.3), compared to those with higher creatinine clearance (300 mg, IQR 291.6–333.3) (P < 0.05). The difference in nephrotoxicity can, thus, be attributed to dose.

**Table 4:** Subgroup analysis of Colistin nephrotoxicity

Subgroup	Nephrotoxicity		Difference	95% CI	P value
*DM*	Yes	No	− 0.05	− 0.30 to 0.19	0.674
	11 (36.7)	13 (41.9)			
*Baseline Cr clearance (ml/min)*	≥ 50	< 50	0.40	0.18 to 0.62	0.002
	19 (57.6)	5 (17.9)			
*Age*	≥ 65	< 65	0.12	− 0.13 to 0.37	0.348
	12 (46.2)	8 (38.1)			
*BMI*	≥ 30	< 30	− 0.02	− 0.28 to 0.24	0.885
	8 (38.1)	16 (40.0)			
*APACHE 2 score*	≥ 20	< 20	− 0.22	− 0.47 to 0.02	0.075
	10 (29.4)	14 (51.9)			
*Total daily dose*	≥ 300 mg	< 300 mg	0.42	0.20 to 0.65	0.001
	17 (63.0)	7 (20.6)			

Data are presented as n (%)

There was no effect of diabetes, age and BMI on Colistin nephrotoxicity. A striking observation in our study was the presence of higher incidence of renal failure in patients with lower APACHE 2 score (14 of 27, 51.9%) compared to higher APACHE 2 score (10 of 34, 29.4%), although this was not statistically significant (P = 0.075). It is very unlikely that greater degree of sickness in any patient will confer any degree of protection against Colistin nephrotoxicity. When we did post hoc analysis of these patients, we found that patients in higher APACHE group received lower Colistin dose (233.3 mg, IQR 191.6–283.3) compared to patients in lower APACHE group (300 mg, IQR 166.7–308.3). Bivariate analysis of risk factors for Colistin induced nephrotoxicity has been shown in Table 5.

**Table 5:** Predictors of Colistin nephrotoxicity

Variable	Bivariate analysis		
	OR	95% CI	P value
BMI ≥ 30	0.92	0.31 to 2.73	0.885
DM (yes)	0.80	0.29 to 2.24	0.802
Age ≥ 65	1.64	0.58 to 4.65	0.350
Daily dose ≥ 300 mg/day	6.56	2.10 to 20.52	0.001
APACHE ≥ 20	0.39	0.13 to 1.11	0.387
Baseline creatinine clearance < 50 ml/min	0.16	0.05 to 0.53	0.003

Among patients who developed renal failure with Colistin, 75% (18 of 24) had recovery from renal failure by 1 week. On subgroup analysis, there was no significant effect of Age, BMI, dose, or baseline creatinine clearance on reversal of Colistin toxicity (Table 6).

**Table 6:** Subgroup analysis of reversal of Colistin nephrotoxicity

Subgroup	Nephrotoxicity		Difference	95% CI	P value
DM	Yes	No	0.13	− 0.21 to 0.46	0.478
	9 (81.8)	9 (69.2)			
Baseline creatinine clearance (ml/min)	≥ 50	< 50	0.19	− 0.28 to 0.66	0.384
	15 (78.9)	3 (60)			
Age	≥ 65	< 65	0	− 0.35 to 0.35	1.000
	9 (75.0)	9 (75.0)			
BMI	≥ 30	< 30	− 0.52	− 0.84 to (− 0.21)	0.064
	8 (100)	10 (62.5)			
Total daily dose	≥ 300 mg	< 300 mg	0.05	− 0.34 to 0.44	0.795
	13 (76.5)	5 (71.4)			

Out of 51 patients in PMB group, only 6 (11.8%) developed nephrotoxicity. Interestingly, out of these 6 patients, 5 patients (83.3%) had baseline renal dysfunction, i.e. creatinine clearance < 50 ml/min. There was no effect of any other factor on Polymyxin B nephrotoxicity. There was recovery from nephrotoxicity in 83.3% (5 of 6) patients by 1 week.

## Discussion

In our study, use of Colistin was associated with significantly higher nephrotoxicity as compared to Polymyxin B. The patients in two groups of our study were similar in baseline demographics, comorbidities, APACHE 2 score, baseline creatinine clearance. And thus, it is unlikely that difference in any of these factors affected the results.

The incidence of nephrotoxicity in our study was 39.3% with Colistin and 11.8% with Polymyxin B. Nephrotoxicity was defined as greater than or equal to two-fold rise in serum creatinine value or 50% decline in estimated creatinine clearance. This corresponds to injury (I) and failure (F) classes of RIFLE classification. Published literature has shown significant variability in nephrotoxicity rates with polymyxin drugs. Recent studies [4–8] have reported renal failure rates of 17–60% with Colistin. A most recent prospective study has shown renal failure rates of 16.9% with Colistin [9].

There are multiple reasons for these discrepancies in the reported rates of nephrotoxicity. Different studies have used different criteria to define nephrotoxicity. Even when RIFLE criteria were used, ‘risk (R)’ category was included as part of renal injury in some studies [4, 5, 7, 8] while it was excluded in other studies [6]. In a recent study, only failure (F) category was used to define renal failure [9]. Another source of discrepancy is the presence of additional confounding factors contributing to renal failure [6, 9]. In the recent study by Rigatto et al. 84% of patients in Colistin group and 71% of patients in Polymyxin B group received a second nephrotoxic drug (aminoglycosides, vancomycin, intravenous contrast) [9]. Similarly, in the study by Dalfino et al. 50% patients in Colistin group additionally received aminoglycosides [6].

Different dosing regimens have been used in different studies, and are likely to have contributed to variations in nephrotoxicity rates. As per most recent recommendations based on pharmacokinetic principles, Colistin is recommended to be administered as a loading dose followed 12 h later as maintenance doses [2]. Different studies have used Colistin in different dosing schedules. Patients within a study have received different Colistin doses. Colistin loading has not been used in many studies [4, 6, 7]. In the study by Rigatto et al. only 27% patients in Colistin group received a loading dose [9]. Similarly, there are recommendations for no dose adjustment for Polymyxin B according to creatinine clearance [3]. The low rates of nephrotoxicity with Polymyxin B in previous studies may be, in part, due to lower doses used in patients with reduced creatinine clearance, with consequent reduced renal exposure to the drug [4, 5, 9, 13].

In our study, a uniform dosing schedule for Colistin and Polymyxin B was used (Table 1). All patients in both groups received a loading dose. Colistin dosing was based on ideal body weight, and creatinine clearance. Dosing of Polymyxin B was based on actual body weight. There was no dose modification for reduced creatinine clearance in PMB group.

One of the strength of our study was elimination of all possible confounding factors that can contribute to renal failure. We excluded patients from the study if they received any nephrotoxic drug. We also excluded patients who received diuretic infusion after initiation of Colistin or Polymyxin B, because diuretics have shown to potentiate polymyxin nephrotoxicity [14, 15]. Despite an adequate control of various covariates, some degree of AKI may occur because of any residual confounding factor in critically ill patients. The ‘risk (R)’ category of RIFLE classification is most affected by this confounding, hence, we excluded ‘R’ category from our definition of nephrotoxicity.

In our study, 14.8% patients in Colistin group required hemodialysis and drug discontinuation, compared to 2% patients in Polymyxin B group. Compared to earlier studies which reported onset of renal failure at 6–7 days, we observed that nephrotoxicity developed consistently early in our patients [5, 6]. The median onset of renal failure in Colistin was 4 days (mean 3.8 ± 0.8), and with Polymyxin B was 4 (mean 4.2 ± 0.7) days.

Colistin and Polymyxin B cause renal failure by increasing the tubular epithelial cell membrane permeability, which leads to ions and water influx leading to cell lysis [16]. The difference in nephrotoxicity can be explained by their different pharmacokinetic profiles. Colistin has a ‘flat’ concentration vs time profile while Polymyxin B has larger peak-to-trough variations. This results in increased exposure of renal tubular cells to Colistin leading to nephrotoxicity [17]. Other possible contributors to Colistin toxicity are the multiple compounds which are formed during conversion of prodrug CMS to Colistin.

Subgroup analysis of COL group was done to analyse factors associated with Colistin nephrotoxicity. Use of higher daily dose (≥ 9 MIU or 300 mg) of Colistin was associated with significant risk of renal failure (OR 6.56, 95% CI 2.10–20.52, P = 0.001). Similar findings were reported in two retrospective studies [14, 15]. We also found that risk of renal failure was less in patients with lower creatinine clearance (OR 0.16, 95% CI 0.05–0.53, P = 0.003) (Table 4), and this was likely due to lower doses used in these patients. We do not feel that the loading dose of Colistin is responsible for its toxicity, because patients in lower (daily) Colistin dose group received similar loading doses. Some studies have found an association between total cumulative dose of Colistin and its toxicity [18, 19]. However, in our study, the vast majority of nephrotoxicity occurred with first 4 days of therapy, and thus no association between cumulative dose and toxicity was observed.

We, thus, found that Colistin nephrotoxicity was related to amount of daily maintenance dose and was not related to loading dose or cumulative total dose. Higher maintenance doses of Colistin have been recommended to optimize serum concentrations, and thus antibiotic efficacy [2]. Does this mean that present recommendations of maintenance dosing need further optimization to reduce its nephrotoxicity? Larger pharmacokinetic—based studies are needed to answer this question.

19 patients in Colistin group (out of 61) had urinary tract infection (UTI). On analysing these 19 patients, we found that 2 patients had upper urinary tract infections (pyelonephritis), while the remaining 17 patients had lower urinary tract infections. Presence of upper urinary tract infection can, per se, lead to kidney injury. However, we feel that it was not a significant confounding factor in our findings, because of following reasons. First, we excluded patients who had fluctuating (improving or worsening) renal parameters prior to starting Colistin or Polymyxin B. Second, even after starting Polymyxin B or Colistin, nephrotoxicity criteria were required to be fulfilled for at least 2 consecutive determinations 24 h apart, 48 h after starting the drug. Finally, “risk” category of RIFLE classification was excluded from our definition of nephrotoxicity, which is most affected by any such confounding factor.

The reversal of nephrotoxicity has not been reported in most of the earlier studies. Dewan et al. reported that all patients who developed renal failure had reversal by 8 days and no patient required dialysis [8]. In our study, a significant number of patients (75%) who developed Colistin toxicity had a reversal by 1 week. We did not find any significant factor contributing to this reversal.

In the polymyxin group, a striking observation was that most patients who developed nephrotoxicity were those who had a baseline renal dysfunction (creatinine clearance less than 50 ml/min). Since, polymyxin dosing is not affected by creatinine clearance, these patients had also received similar doses as patients with normal creatinine clearance [3]. Sandri et al. found that renal clearance is a minor elimination pathway in case of Polymyxin B, and hence they recommended no dose reduction in renal dysfunction. But they observed that reduced renal elimination is basically due to extensive tubular reabsorption, and this tubular reabsorption contributes to its nephrotoxicity. Thus, it is likely that, in a patient with lower creatinine clearance, the ‘fewer’ functioning nephrons are exposed to relatively ‘large’ dose of Polymyxin B, when no dose reduction is done. In other words, it is likely that, unlike Colistin, where there is an overlap between therapeutic doses and toxicity doses, in case of Polymyxin B, there is a gap between the two. Toxicity thus manifests only in patients with compromised renal function, who get exposed to “significantly” large doses. Does this mean that polymyxin dosing also needs reduction in renal failure? We need larger prospective studies on Polymyxin B nephrotoxicity to answer this question.

Our study was limited by smaller number of patients in two groups. Some of our observations might have been affected by smaller group size. Also, we did not measure serum Colistin levels, which would have given more insights into its pharmacokinetics and its relationship with nephrotoxicity. Finally, we followed up our patients for 1 week after stopping the drug. Extending the follow up period to 6–8 weeks could have given better understanding of recovery of nephrotoxicity.

To conclude, our study showed that Colistin is more nephrotoxic than Polymyxin B. We also observed that dose is the principal factor contributing to Colistin nephrotoxicity. Nephrotoxicity is mostly mild to moderate and reversible. Larger studies are needed to confirm our observations.

## Abbreviations

COL: Colistin
PMB: Polymyxin B
BMI: body mass index
CKD: chronic kidney disease
IQR: inter quartile range
MDR: multi-drug resistant
ADQI: acute dialysis quality initiative
AKI: acute kidney injury
APACHE: acute physiology and chronic health evaluation
